# Supplementary material for: SyNDI: synchronous network data integration framework
Source: BMC Bioinformatics. 2018 Nov 6;19:403. doi: 10.1186/s12859-018-2426-5 (PMC6219086; doi:10.1186/s12859-018-2426-5)
Supplement: Supplementary file 5 — Files of the example presented in Additional file 4. This additional file is a zip package that contains all files used in Additional file 4. Detailed descriptions of each of these files are presented in a README file included in this zip package. (GZ 30411 kb) [file 12859_2018_2426_MOESM5_ESM.gz › SyNDITools/Meme2Fimo/meme_4.9.0_4.tar.gz!/meme_4.9.0/website/cgi-bin/spamo.tmpl]

Spamo - Submission form


Select a file of FASTA formatted
DNA sequences or paste in actual FASTA formatted DNA sequences to analyse motif spacings.

See the sample sequences
to get an idea of input that works well for SpaMo.

[ close ]

Spamo expects a DNA motif file.
Only the first motif will be used. See the sample motif file.

[ close ]

SpaMo can use databases installed on the server or search a user
uploaded database in MEME DNA motif
format by selecting the "Upload Your Own Database" option. For more
information browse the
supported databases.

[ close ]

An uploaded database is a normal MEME DNA motif file with lots of
motifs. The uploaded database must be smaller than 5MB.

[ close ]

The email is used to notify you of your results.

[ close ]

The job description will be included in the notification email you
receive and in the SpaMo output.

[ close ]

This writes out a text file for every primary and secondary motif which
has a significant spacing. The text file contains fields which from left
to right are:

1. A portion of the sequence in lower case with the motif
   matches in upper case.
2. The position of the secondary match within the sequence
   portion.
3. The fragment of the sequence which matched the primary
   motif.
4. The strand which matched the primary motif.
5. The fragment of the sequence which matched the secondary
   motif.
6. The strand which matched the secondary motif.
7. Secondary site is on the same (s) or opposite (o) strand
   to the primary site.
8. Secondary site is upstream (u) or downstream (d) of the
   primary site.
9. The gap between the primary and secondary site.
10. The name of the sequence.

[ close ]

|  |  |
| --- | --- |
|  | MEME Suite  The MEME Suite web application requires the use of JavaScript  Javascript doesn't seem to be available on your browser.  Version  Use this form to find enriched spacings between a **primary motif** and each motif in database of **secondary motifs** for a set of sequences. Typically, the sequences will be centered on ChIP-seq peaks.  Data Submission Form Discover enriched spacings between motifs.  Input the sequences  Enter DNA sequences in which you want to analyse motif spacings  or paste the sequences   Input the primary motif  Enter a motif to use as the primary   or checked="checked"> use submitted motif   Input the secondary motifs  Upload Your Own Database  Input secondary motifs to upload  Select a MEME motif database file.   Input job queue details  Enter your email address.  Re-enter your email address.  Advanced Options  Advanced Options  Dump matches to file |
